# Acute Kidney Injury and Hyperkalemia With Precarious Electrocardiographic Changes Caused by Concurrent Use of Telmisartan and Diclofenac

**DOI:** 10.7759/cureus.9858

**Published:** 2020-08-18

**Authors:** Saurabh Gaba, Gautam Jesrani, Samiksha Gupta, Monica Gupta

**Affiliations:** 1 General Medicine, Government Medical College and Hospital, Chandigarh, IND

**Keywords:** aki, acute kidney injury, telmisartan, diclofenac, drug induced, dialysis, hyperkalemia, ecg

## Abstract

A 70-year-old hypertensive man was prescribed telmisartan for control of blood pressure. He concurrently took over-the-counter diclofenac for back pain. Few days later, he presented to the casualty after syncopal episodes. He was found to have acute kidney injury and elevated potassium of 6.6 mmol/L with junctional bradycardia on electrocardiogram (ECG). Medical measures were instituted for hyperkalemia and sinus rhythm was restored, but peaked T waves were still present in the precordial leads. Hemodialysis was done, and antihypertensive therapy was changed on discharge.

## Introduction

Hyperkalemia is a medical emergency due to its potential to cause life-threatening arrhythmias. The risk depends on the potassium level and the time period over which it develops [1]. The etiologic factors are diverse and include renal failure, drugs, states of increased cell turnover and certain endocrine disturbances. Herein, a case of hyperkalemia resulting from concurrent use of telmisartan and diclofenac leading to a catastrophic clinical scenario is presented.

## Case presentation

**Figure 1:**
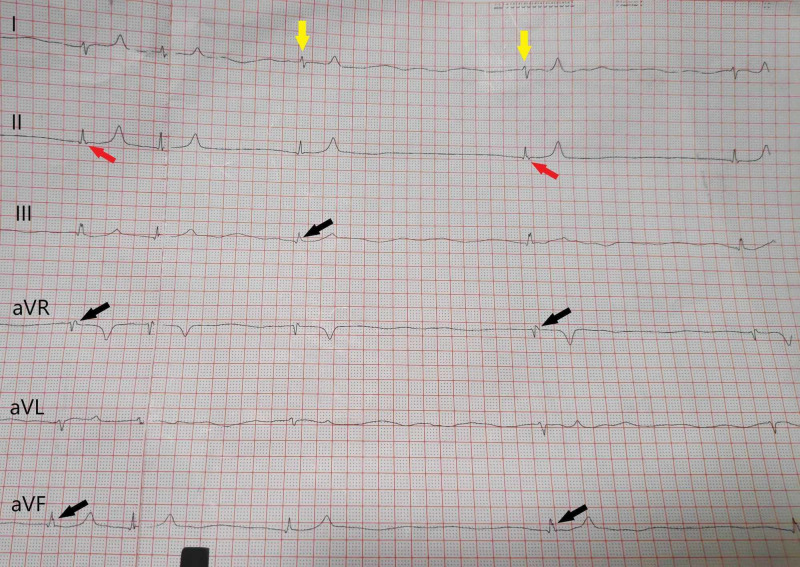
The initial electrocardiogram showing junctional bradycardia. The QRS complexes (yellow arrows) are narrow (<120 ms). The P waves are retrograde as they occur after the QRS complex. They are either upright (black arrows) or inverted (red arrows).

**Figure 2:**
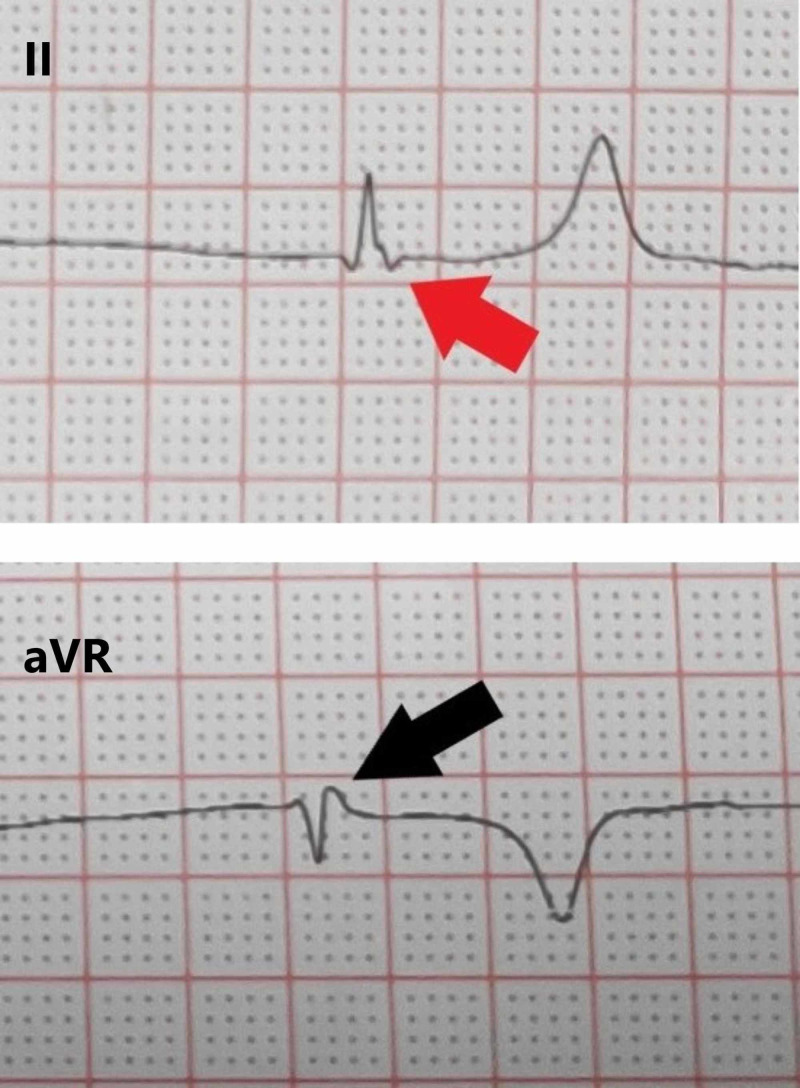
Enlarged complexes from the initial electrocardiogram showing junctional bradycardia. The retrograde P waves are inverted (red arrow) in lead II and upright (black arrow) in lead aVR.

A 70-year-old male was prescribed telmisartan 40 mg a day to achieve the target blood pressure (BP), since his BP was not well controlled on amlodipine that he had been taking for the previous few months. He had no other significant past medical history. His serum creatinine and potassium, measured a few weeks earlier, were 1.3 mg/dL and 4.1 mmol/L, respectively. After starting telmisartan, he also took over-the-counter diclofenac 50 mg, up to three tablets a day, for his back pain. He presented to the casualty after a week on experiencing two syncopal episodes while sitting. They were not accompanied by chest pain, palpitations or breathlessness. There was no history of fever, diarrhea, vomiting, limb weakness or convulsions. On presentation, he had BP of 80/40 mmHg and pulse of 20 beats per minute. His electrocardiogram (ECG) showed junctional bradycardia (Figures 1, 2).

**Figure 3:**
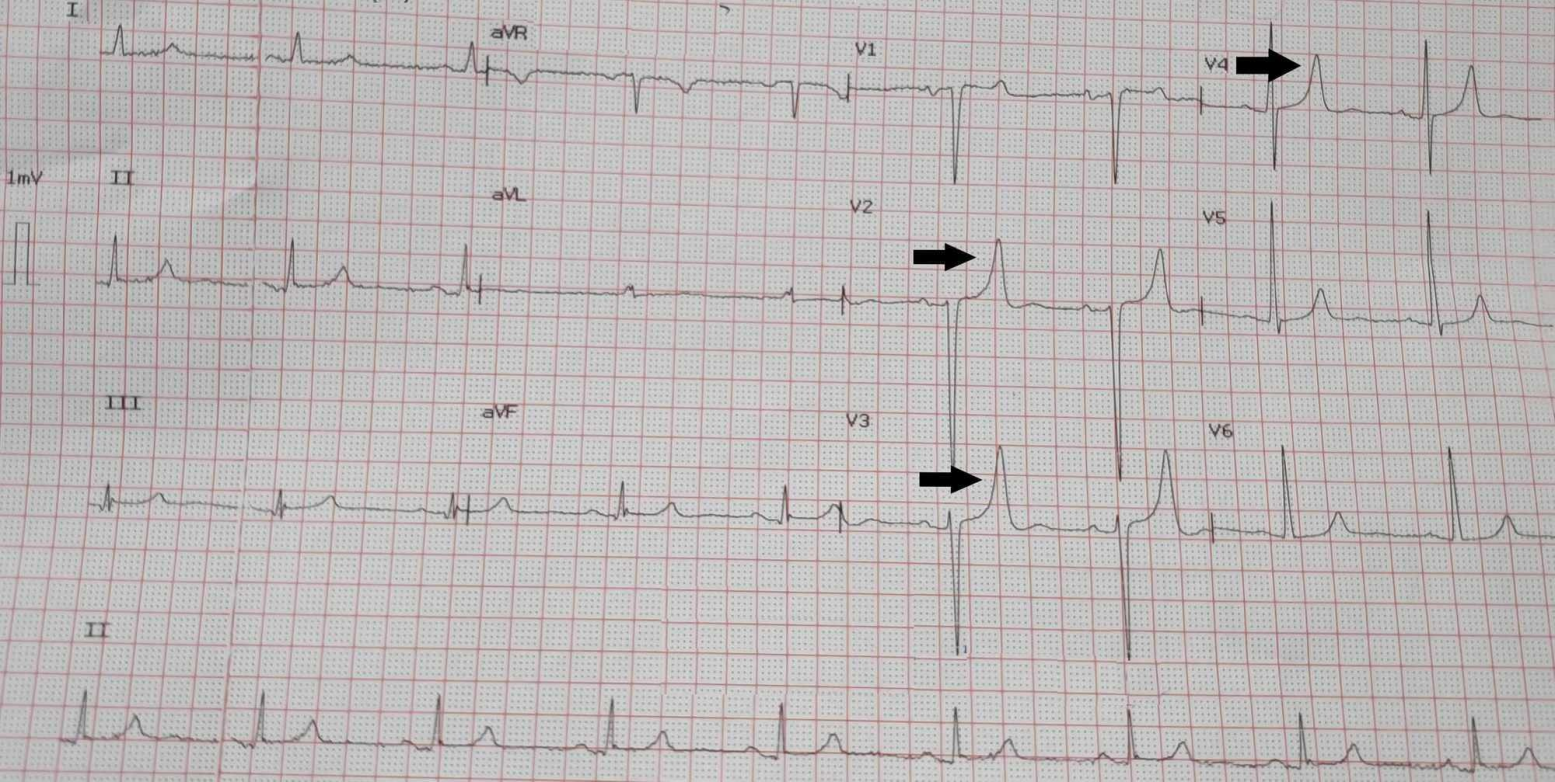
The electrocardiogram showing sinus rhythm with peaked T waves (black arrows) after institution of medical measures for control of hyperkalemia.

The cardiology team was informed and preparations were made for temporary pacemaker placement. Meanwhile, a sample was sent for venous blood gas analysis for quick assessment of electrolytes. The potassium was found to be elevated to 6.6 mmol/L. The patient was administered intravenous calcium gluconate and dextrose with insulin, and nebulization was done with salbutamol. Within seconds, sinus rhythm was restored, albeit with peaked T waves (Figure 3). 

One session of hemodialysis was done, and the clinical course was uneventful thereafter. Telmisartan was substituted with hydrochlorthiazide. The serum creatinine and potassium values are mentioned in Table 1. No abnormality was found on urinalysis and ultrasound of the kidneys. 

**Table 1:** Trend of creatinine and potassium during the hospital stay.

Investigation	Day 1 (before hemodialysis)	Day 1 (after hemodialysis)	Day 6
Creatinine (mg/dL)	2.9	1.7	1.6
Potassium (mmol/L)	6.6	4.0	3.7

## Discussion

The serum potassium levels are tightly regulated in the range of 3.5-5.5 mmol/L by a complex interplay of urinary excretion, gastrointestinal loss, transcelluar shifts and dietary intake. The causes of hyperkalemia are mentioned in Table 2 [2,3]. In addition, pseudohyperkalemia can occur due to the release of potassium from cells in the presence of hemolysis, traumatic venipuncture, prolonged clenching of fist or when the vial with blood sample is shaken.

**Table 2:** Etiology of hyperkalemia. ACEI, angiotensin-converting enzyme inhibitor; ARB, angiotensin receptor blocker; NSAID, non-steroidal anti-inflammatory drug.

Category	Causes
Decreased renal loss	Acute kidney injury, chronic kidney disease, type IV renal tubular acidosis, adrenal insufficiency, drugs (ACEI, ARB, NSAID, heparin, cyclosporin, tacrolimus, spironolactone, eplerenone)
Increased load	High potassium diet, hemolysis, tumor lysis syndrome, rhabdomyolysis, blood transfusions, gastrointestinal hemorrhage
Extracellular shift	Drugs (digoxin, non-selective beta blockers), hyperglycemia, hyperkalemic periodic paralysis, strenuous exercise

Angiotensin-converting enzyme inhibitors (ACEIs) and angiotensin receptor blockers (ARBs) decrease angiotensin II-mediated constriction of the efferent arterioles in renal glomeruli, leading to reduced glomerular filtration rate (GFR) [4]. In some patients, acute kidney injury (AKI) can be precipitated. Patients with a pre-existing renal disease, renovascular disease or dehydration are more susceptible. Apart from being related to AKI, hyperkalemia can also occur secondary to decreased aldosterone level and retention of potassium in the distal renal tubules. It is recommended to monitor potassium levels after starting ACEI/ARB or after increasing their dose. Conversely, non-steroidal anti-inflammatory drugs (NSAIDs) inhibit prostaglandin-mediated dilatation of afferent arterioles, predisposing the patient to AKI [5]. AKI can also result from NSAID-induced tubulointerstitial nephritis. The concurrent use of these drugs, as in the patient under consideration in this report, places the patient at a very high risk of AKI and hyperkalemia.

Postassium plays a key role in cardiac impulse generation and propagation. Hyperkalemia leads to decrease in the myocyte resting membrane potential, prolongation of membrane depolarization time and shortening of the repolarization time [6]. These electrophysiological perturbations result in characteristic ECG changes (Table 3) [7]. However, the relationship between potassium level and ECG finding is not uniform, and the sequential changes may not necessarily be seen at the levels mentioned. Acute rises in potassium produce more pronounced ECG changes. Fatal rhythms like ventricular tachycardia, ventricular fibrillation, complete heart block and asystole can occur precipitously, giving little time for treatment. In a study, 54% of the patients with serum potassium more than 6 mmol/L did not have the specific ECG changes consistent with hyperkalemia [8]. Cases with potassium more than 8 mmol/L without the typical ECG changes have also been reported. One of the earliest and easily identifiable sign of hyperkalemia is the presence of peaked T waves in precordial leads (V2-V4) and limb leads (II and III). They have a narrow base, in contrast to the broad-based peaked T waves seen in early myocardial infarction [7].

**Table 3:** The electrocardiographic findings in hyperkalemia.

Potassium level (mmol/L)	ECG finding
5.5–6.5	Peaked T waves, short PR interval, short QT interval, sinus tachycardia or bradycardia
6.5–8.0	Wide QRS complex, prolonged PR interval, sinus tachycardia or bradycardia, heart blocks
8.0–9.0	Wide QRS complex, absent P waves (mimicking ventricular tachycardia), heart blocks, ventricular tachycardia
>9.0	Junctional rhythm, blending of P and T waves with the wide QRS complex (sine wave pattern), ventricular tachycardia, ventricular fibrillation, asystole

The treatment of hyperkalemia is straightforward and very effective. In most of the cases, immediate correction assumes importance over addressing the underlying cause. The myocyte electrophysiological changes can be temporarily antagonized by administration of intravenous 10% calcium gluconate over three minutes under continuous cardiac monitoring [9]. Its action starts within a minute and lasts for an hour. Immediate reduction in serum potassium level is achieved by promoting its influx into cells by salbutamol nebulization and intravenous insulin [10]. Typically, 10 units of regular insulin are given in 25% or 50% dextrose solution. If blood glucose is more than 250 mg/dL, then dextrose is not needed. Both salbutamol and insulin work by potentiating the action of sodium-potassium adenosine triphosphatase (Na/K-ATPase) pump. The effect of insulin is much more potent and clinically important than salbutamol, and it can lower serum potassium by 1 mmol/L within 30 minutes [11]. The reduction is temporary as total body potassium is not altered. Mild reduction over several hours can also be achieved by bicarbonate infusion, but its use is restricted to patients with acidemia [12]. Finally, the fastest and most effective to reduce total body potassium is hemodialysis. For patients with life-threatening arrhythmias, early hemodialysis is essential to avoid cardiac arrest. The medical measures should always be instituted first as access to hemodialysis can take time [9]. The role of ion-exchange resins, such as sodium polystyrene sulfonate and patiromer, in emergency setting is limited as they take several hours or days to produce effect. They enhance potassium elimination by gastrointestinal route and are used in conjunction with laxatives to prevent constipation [9].

The common causes of junctional rhythm include sick sinus syndrome, coronary artery disease, drugs (antiarrhythmics, beta blockers, digoxin), hypoxia, ischemic heart disease and hyperkalemia [13]. It can also occur in athletes due to increased vagal tone. Treatment is not always required and depends on the underlying morbidity, heart rate and evidence of cardiac decompensation. The modalities include withdrawal of the implicated drug, correction of potassium, reversal of hypoxia, management of ischemic heart disease and placement of a permanent cardiac pacemaker.

## Conclusions

This case emphasizes the hazards of concurrent use of drugs having potential to cause AKI and hyperkalemia. The patient took an over-the-counter NSAID, without knowledge of his physician, in addition to an ARB that was prescribed to him. This led to hyperkalemia with dangerous ECG changes that necessitated urgent hemodialysis.
